# EFFECTS: an expanded access program of everolimus for patients with subependymal giant cell astrocytoma associated with tuberous sclerosis complex

**DOI:** 10.1186/s12883-016-0658-4

**Published:** 2016-08-08

**Authors:** Andras Fogarasi, Liesbeth De Waele, Gabriella Bartalini, Sergiusz Jozwiak, Nicola Laforgia, Helene Verhelst, Borivoj Petrak, Jean-Michel Pedespan, Olaf Witt, Ramon Castellana, Stefania Crippa, Gabriella Gislimberti, Zsuzsanna Gyorsok

**Affiliations:** 1 Neurology Department, Bethesda Children’s Hospital, Bethesda Street 3, H-1146 Budapest, Hungary; 2 Gasthuisberg University Hospitals Leuven, Leuven, Belgium; 3 Pediatric Unit, University Hospital, Siena, Italy; 4 Children’s Memorial Health Institute, Warsaw, Poland; 5 Department of Pediatric Neurology, Medical University of Warsaw, Warsaw, Poland; 6 Neonatology and NICU Section, DIMO, University of Bari, Bari, Italy; 7 Ghent University Hospital, Ghent, Belgium; 8 Motol University Hospital, Prague, Czech Republic; 9 CHU Pellegrin–Hôpital des Enfants, Bordeaux, France; 10 German Cancer Research Center and Section of Pediatric Brain Tumors, University Hospital, Heidelberg, Germany; 11 Novartis Farmacéutica SA, Barcelona, Spain; 12 Novartis Farma S.p.A., Origgio, Italy; 13 National Institute of Neurosciences, Budapest, Hungary

**Keywords:** Tuberous sclerosis, Expanded access program, Everolimus, Subependymal giant cell astrocytoma

## Abstract

**Background:**

Everolimus, a mammalian target of rapamycin (mTOR) inhibitor, has been shown to be effective and safe in the treatment of subependymal giant cell astrocytoma (SEGA) associated with tuberous sclerosis complex (TSC). The Everolimus For Fast Expanded aCcess in TSC SEGA (EFFECTS) study was designed to provide everolimus access to patients with SEGA associated with TSC and to mainly assess the safety and also efficacy of everolimus in a real-world setting.

**Methods:**

EFFECTS was a phase 3b, open-label, noncomparative, multicenter, expanded access study. Eligible patients were ≥ 3 years of age, with a definite diagnosis of TSC, and with at least one SEGA lesion identified by MRI or CT scan. Patients received once daily everolimus (dose adjusted to attain a trough level of 5-15 ng/mL). Safety evaluation was the primary objective and included collection of adverse events (AEs) and serious AEs, with their severity and relationship to everolimus. Efficacy evaluation, which was the secondary objective, was based on the best overall response as per medical judgment.

**Results:**

Of the 120 patients enrolled, 100 (83.3 %) completed the study. Median age of patients was 11 years (range, 1-47). Median daily dose of everolimus was 5.82 mg (range, 2.0–11.8). Median duration of exposure was 56.5 weeks (range, 0.3–130). The overall incidence of AEs was 74.2 %. Aphthous stomatitis (18 [15.0 %]), pyrexia (18 [15.0 %]), bronchitis (11 [9.2 %]), and stomatitis (10 [8.3 %]) were the most common AEs reported. Overall, 25 patients had grade 3 AEs; most frequent was stomatitis (4 [3.3 %]). Grade 4 AEs were reported in three (2.5 %) patients. A total of 62 (51.7 %) patients had suspected drug-related AEs, of which 15 (12.5 %) were of grade 3 or 4. In eight (6.7 %) patients, AEs led to drug discontinuation. With regard to efficacy, 81 (67.5 %) patients had a partial response, 35 (29.2 %) had a stable disease, and one (0.8 %) had progressive disease. The response was unknown in three (2.5 %) patients.

**Conclusion:**

This study confirms the acceptable safety profile of everolimus in patients with SEGA associated with TSC in a real-world setting. The results further support the efficacy of everolimus in the treatment of SEGA associated with TSC. (EudraCT: 2010-022583-13)

## Background

Tuberous sclerosis complex (TSC) is a genetic disorder that is associated with the development of benign tumors in multiple organs throughout the body [1–3]. The birth incidence of TSC is about one in 6000 individuals [2]. Although any organ system can be affected, organs commonly involved include the brain, kidneys, skin, lungs, heart, eyes, and liver. Patients may present with varied manifestations depending on the organs involved [1–3]. TSC is diagnosed based on the presence of certain major and minor criteria. The criteria for diagnosis have been recently revised at the 2012 International TSC Consensus Conference [4].

The brain is the most commonly affected organ in patients with TSC [5]. Most common brain lesions include cortical and subcortical tubers, subependymal nodules, and subependymal giant cell astrocytomas (SEGAs) [1–3]. SEGAs usually develop in childhood or adolescence and are seen in up to 20 % of the patients with TSC. These tumors are generally located near the foramen of Monro [5]. Although SEGAs usually grow slowly, they have the potential to enlarge enough to cause life-threatening complications including raised intracranial pressure and hydrocephalus [1, 6]. Until recently, surgery was the only option available for the treatment of SEGAs. Surgery can be challenging due to the typical deep location of these lesions. Surgery is also associated with significant morbidity and the incompletely resected SEGAs tend to regrow. In addition, some lesions may not be amenable to surgery [6, 7]. In a study that analyzed 64 SEGA surgeries, risk factors for poor outcome included bilateral tumors, tumors bigger than 2 cm, and children younger than 3 years [8].

TSC is most often caused by mutations in either *TSC1* (hamartin) or *TSC2* (tuberin) gene. Normally, the activation of mammalian target of rapamycin (mTOR) complex 1, which is responsible for cell growth, proliferation, and protein synthesis, is limited by the hamartin-tuberin tumor suppressor complex. Mutations in either *TSC1* or *TSC2* gene lead to constitutive activation of the mTOR complex 1, which in turn leads to development of the hamartomatous lesions seen in patients with TSC [1–4]. Based on this pathophysiological finding, mTOR blockade was explored as a treatment approach for TSC [9, 10]. Everolimus, an mTOR inhibitor, has been evaluated for the treatment of SEGA associated with TSC. In an open-label, phase 1–2 study, everolimus demonstrated significant reduction in the volume of SEGA associated with TSC [10]. The efficacy and safety of everolimus in the treatment of SEGA associated with TSC were confirmed in the randomized, double-blind, phase 3 study — EXamining everolimus In a Study of Tuberous sclerosis complex (EXIST-1) [11]. Based on these results, everolimus was approved by the United States Food and Drug Administration (USFDA; initial accelerated approval in 2010) and European Medicines Agency (EMA; in 2011) for pediatric and adult patients with SEGA associated with TSC [12, 13].

The process of approval had just begun in several European countries when the Everolimus For Fast Expanded aCcess in TSC SEGA (EFFECTS) study was initiated. The purpose of the study was to provide access to everolimus prior to commercial availability in the participating countries and also to further assess the safety and efficacy of everolimus in patients with SEGA associated with TSC.

## Methods

### Study design and patients

EFFECTS was a phase 3b, open-label, noncomparative, multinational, expanded access study of everolimus for the treatment of patients with SEGA associated with TSC. Eligible patients, who were 3 years of age or older, had a definite diagnosis of TSC (as per the modified Gomez criteria) [14], and they had at least one SEGA lesion identified by magnetic resonance imaging (MRI) or computed tomography (CT) scan (according to local requirements by size and/or location). Patients had to be medically stable, with no need of SEGA-related surgery, with no use of an investigational study drug within 30 days prior to enrollment, and they should not have participated in the EXIST-1 study [11]. Written informed consent was obtained from all patients (or their legal representatives) before enrollment. The protocol was approved by an ethics committee at each center, before the first patient was enrolled. The study was conducted in accordance with the principles of Good Clinical Practice, Declaration of Helsinki (2013 version) [15], and all local regulations.

### Treatment

Everolimus was administered as a once-daily oral dose. Starting dose of everolimus was determined by body surface area (BSA). Recommended starting dose was 2.5 mg for BSA ≤ 1.2 m^2^, 5 mg for BSA 1.3 to 2.1 m^2^, and 7.5 mg for BSA ≥  2.2 m^2^. The dose was titrated upward to attain a trough level in the range 5 to 15 ng/mL. Patients were asked to visit the clinic every 2 weeks until the level of everolimus was within the required range of 5 to 15 ng/mL. After stable blood levels of everolimus were reached, patients visited the clinic monthly. The treatment was continued until one of the following conditions occurred: tumor progression (determined by MRI or CT scan); unacceptable toxicity (according to investigators’ medical judgment); death; discontinuation from the study for any other reason; or until the drug became commercially available for SEGA associated with TSC in the respective countries or up to December 31, 2013, whichever occurred earlier. In case the drug was not available in a country as of December 31, 2013, patients were moved to a local transition plan to ensure access to the study medication without any delay in the treatment.

### Assessments

#### Safety assessment

The primary objective of the study was to evaluate the safety of everolimus in patients with SEGA associated with TSC. Adverse events (AEs) were monitored continuously throughout the duration of the study. Grade 3 and 4 AEs, serious AEs (SAEs), and laboratory abnormalities were assessed according to the Common Terminology Criteria for Adverse Events (CTCAE) version 4.0 [16]. Grade 1 or 2 AEs and laboratory or test procedure abnormalities were not reported unless they were clinically significant for the investigator or caused everolimus dose modification or interruption because when compared to standard clinical trials, expanded access program follows a simplified data collection focusing on “key safety aspects”, but beyond only SAE. Each AE was evaluated to determine the following: its severity, its relationship to study drug, its duration, action taken, and whether it was serious. Monitoring of AEs was continued for 4 weeks following the last dose of study treatment.

#### Efficacy assessment

Efficacy was evaluated as a secondary objective. Tumor response and progression were assessed using MRI or CT scans of the brain (volume measurements of SEGA), at the investigators’ discretion. Tumor assessments were performed during screening, at 3, 6, and 12 months, and annually thereafter, as well as at the end of treatment. The overall investigator assessment of best response was collected at the end of the study for the FAS population defined as best radiological and clinical response (radiological response in context with toxicities or other medical observations of the investigator).

#### Pharmacokinetics assessment

The trough (predose) everolimus blood levels were evaluated for all patients at week 2. In addition, trough blood levels were assessed 1 to 2 weeks after the occurrence of any of the following: increase in the dose of everolimus; reduction in the dose of a CYP3A4 or PgP inducer (eg, reduction in anticonvulsant dose); addition of (or increase in the dose of) a CYP3A4 or PgP inhibitor.

### Statistical analyses

Approximately, up to 250 patients were planned to be enrolled based on the expected accrual rates and the planned duration of the trial. Data were summarized with respect to demographic and baseline characteristics, efficacy observations and measurements, safety observations and measurements. Categorical data were presented as frequencies and percentages. Final analyses were performed at the end of the study when all patients had discontinued everolimus (ie, they were moved to commercial drug or to local transition plans by December 31, 2013). No formal statistical tests were conducted. Efficacy population included full analysis set (FAS) defined as patients who received at least one dose of everolimus. Safety population consisted of all patients who received at least one dose of everolimus and had at least one post-baseline safety assessment. In order to evaluate the safety data in the pediatric subpopulation, an additional post hoc analysis by age group (< 18 or ≥  18 years) was made. Statistical analyses were done with SAS software (version 9.3).

## Results

### Overall results

**Fig. 1:**
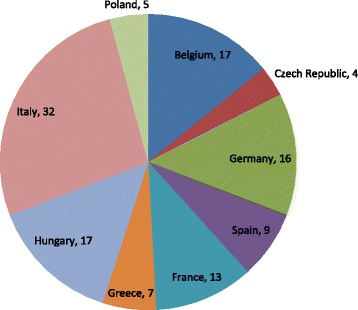
Patients enrolled in EFFECTS

Overall, 120 patients were enrolled from 44 sites in nine countries (Fig. 1) between March 28, 2011 and June 30, 2013. Of the 120 patients, 90 (75 %) patients were <  18 years of age (pediatric subpopulation) and 30 (25 %) patients were ≥  18 years of age. A total of 100 (83.3 %) patients of FAS and 79 (87.8 %) of the pediatric subpopulation completed the study (Table 1), which means that they were either moved to the commercial drug or to the local transition plan by December 31, 2013. Median age of patients was 11 years (range, 1–47; one patient was 1 year old [protocol deviation]) (Table 2).

**Table 1:** Patient disposition

*n* (%)	Everolimus FAS (*N* = 120)	Everolimus pediatric subpopulation (*N* = 90)
Patients		
Completed study	100 (83.3)	79 (87.8)
Discontinued	20 (16.7)	11 (12.2)
Primary reason for premature discontinuation		
Adverse event(s)	8 (6.7) ^a^	4 (4.4)
Other^b^	5 (4.2)	3 (3.3)
Subject withdrew consent	5 (4.2)	2 (2.2)
Administrative problems	1 (0.8)	1 (1.1)
SEGA progression	1 (0.8)	1 (1.1)

*FAS* full analysis set, *SEGA* subependymal giant cell astrocytoma

^a^Includes abnormal laboratory values

^b^Includes investigator’s decision and mother’s decision

**Table 2:** Demographic summary

Demographic characteristics	Everolimus *N* = 120
Age, median (range), years	11.0 (1–47) ^a^
Sex, *n* (%)	
Male	62 (51.7)
Female	58 (48.3)
Race, *n* (%)	
Caucasian	116 (96.7)
Asian	2 (1.7)
Native American	1 (0.8)
Other	1 (0.8)

^a^one patient was 1 year old (protocol deviation)

In addition to SEGA, majority of the patients also had other major neurological features of TSC including subependymal nodule (85 %) and cortical tuber (88.3 %). Other major features frequently seen in this population included hypomelanotic macules (79.2 %), facial angiofibromas or forehead plaques (77.5 %), and renal angiomyolipoma (49.2 %) (Table 3).

**Table 3:** Disease characteristics

Disease characteristics	Everolimus *N* = 120
Time since TSC diagnosis, median (range), years	9.1 (0.2–30.7)
Time since SEGA diagnosis, median (range), years	3.8 (0–24)
Modified Gomez criteria - major features, *n* (%)	
Facial angiofibromas or forehead plaques	93 (77.5)
Non-traumatic ungual or periungual fibroma	18 (15.0)
Hypomelanotic macules (3 or more)	95 (79.2)
Shagreen patch (connective tissue nevus)	47 (39.2)
Multiple retinal nodular hamartomas	23 (19.2)
Cortical tuber	106 (88.3)
Subependymal nodule	102 (85.0)
Subependymal giant cell astrocytoma	120 (100.0)
Cardiac rhabdomyoma, single or multiple	55 (45.8)
Lymphangioleiomyomatosis	2 (1.7)
Renal angiomyolipoma	59 (49.2)
Modified Gomez criteria - minor features, *n* (%)	
Multiple, randomly distributed pits in dental enamel	6 (5.0)
Hamartomatous rectal polyps	0 (0.0)
Bone cysts	2 (1.7)
Cerebral white matter radial migration lines	16 (13.3)
Gingival fibromas	6 (5.0)
Non-renal hamartoma	3 (2.5)
Retinal achromic patch	4 (3.3)
'Confetti' skin lesions	15 (12.5)
Multiple renal cysts	19 (15.8)

*SEGA* subependymal giant cell astrocytoma, *TSC* tuberous sclerosis complex

### Treatment exposure

Median daily dose of everolimus was 5.82 mg (range, 2.0–11.8 mg), including days of temporary interruption of the study drug. Median duration of exposure for all the 120 patients was 56.5 weeks (range, 0.3–130 weeks). Everolimus was administered to 57.5 % of patients for at least 48 weeks and 31.7 % for at least 88 weeks.

### Safety

Overall, 89 (74.2 %) patients had at least one AE. The most common AEs were aphthous stomatitis, pyrexia, bronchitis, and stomatitis (Table 4). Grade 3 and 4 AEs were reported in 25 (20.8 %) and three (2.5 %) patients, respectively. The most frequent grade 3 AE was stomatitis (4 patients, 3.3 %). All the other AEs of grade 3 or 4 were reported in ≤  2 patients. Grade 4 AEs included acute respiratory failure, gastroenteritis, increased gamma-glutamyltransferase (ƴ-GT), near drowning, and aspiration pneumonia. Amenorrhea was reported in three patients (2.5 % of safety population; 12.5 % [3 of 24] of females of childbearing potential). A total of 62 (51.7 %) patients had suspected drug-related AEs (Table 5) (14 [11.7 %] of grade 3 and 1 [0.8 %] of grade 4). The suspected drug-related AEs reported in ≥  5 % of patients were aphthous stomatitis (18 patients, 15 %), stomatitis (9 patients, 7.5 %), pyrexia (8 patients, 6.7 %), and mouth ulceration (6 patients, 5 %).

**Table 4:** Adverse events in >  3 % of patients

AEs, *n* (%)	Safety population (*N* = 120)		Pediatric subpopulation (*N* = 90)	
	All Grades	Grade 3 or 4	All Grades	Grade 3 or 4
Patients with any AE(s)	89 (74.2)	28 (23.3)	67 (74.4)	23 (25.5)
Preferred term				
Aphthous stomatitis	18 (15.0)	2 (1.7)	13 (14.4)	1 (1.1)
Pyrexia	18 (15.0)	1 (0.8)	16 (17.8)	1 (1.1)
Bronchitis	11 (9.2)	1 (0.8)	11 (12.2)	1 (1.1)
Stomatitis	10 (8.3)	4 (3.3)	7 (7.8)	4 (4.4)
Cough	6 (5.0)	-	5 (5.6)	-
Diarrhea	6 (5.0)	1 (0.8)	5 (5.6)	1 (1.1)
Headache	6 (5.0)	1 (0.8)	3 (3.3)	1 (1.1)
Mouth ulceration	6 (5.0)	-	5 (5.6)	-
Sinusitis	6 (5.0)	1 (0.8)	6 (6.7)	1 (1.1)
Abdominal pain	5 (4.2)	-	3 (3.3)	-
Blood creatine phosphokinase increased	5 (4.2)	1 (0.8)	3 (3.3)	1 (1.1)
Gastroenteritis	5 (4.2)	1 (0.8)	4 (4.4)	1 (1.1)
Hypercholesterolemia	5 (4.2)	-	-	-
Pharyngitis	5 (4.2)	-	5 (5.6)	-
Pneumonia	5 (4.2)	-	5 (5.6)	-
Pneumonitis	5 (4.2)	-	5 (5.6)	-
Upper respiratory tract infection	5 (4.2)	-	4 (4.4)	-
Menstruation irregular	4 (3.3) ^a^	-	3 (3.3)	-
Nasopharyngitis	4 (3.3)	-	4 (4.4)	-
Otitis media	4 (3.3)	-	4 (4.4)	-
Urinary tract infection	4 (3.3)	-	-	-

*AE* adverse event

^a^This percentage includes all patients as the denominator. If we consider the females with child bearing potential (*n* = 24), 16.6 % of patients reported menstrual irregular events

**Table 5:** Deaths, serious adverse events, and adverse events leading to permanent discontinuation of study drug (safety population)

AEs, *n* (%)	Everolimus *N* = 120
Patients with any AE(s)	89 (74.2)
Death	0 (0.0)
SAE(s)	32 (26.7)
Discontinuation due to AE(s) ^a^	8 (6.7)
AE(s) causing dose adjustment or study drug interruption	36 (30.0)
CTC Grade 3 or 4	28 (23.3)
Leading to hospitalization/prolonged hospitalization	29 (24.2)
Suspected to be drug related	62 (51.7)

*AE* adverse event, *CTC* common terminology criteria, *SAE* serious adverse event

^a^Includes abnormal laboratory values

In the pediatric subpopulation, 67 (74.4 %) patients experienced AEs (Table 4). Of these, 21 (23.3 %) patients had grade 3 AEs and 2 (2.2 %) patients had grade 4 AEs. The most frequent AE (reported in >  2 % of patients) of grade 3 was stomatitis (4 patients, 4.4 %). All the other AEs of grade 3 or 4 were reported in one patient each. Grade 4 AEs included acute respiratory failure, gastroenteritis, near drowning, and aspiration pneumonia. A total of 45 (50 %) patients had suspected drug-related AEs; those reported in ≥  5 % of patients were aphthous stomatitis (13 patients, 14.4 %), stomatitis (7 patients, 7.8 %), pyrexia (7 patients, 7.8 %), mouth ulceration (5 patients, 5.6 %), and pneumonitis (5 patients, 5.6 %). Twelve (13.3 %) patients were reported to have suspected drug-related AEs of grade 3; the only suspected drug-related AE of grade 3 reported in more than one patient was stomatitis (4 patients, 4.4 %); no patient reported a suspected drug-related AE of grade 4.

Overall, AEs requiring study drug dose adjustment or interruption were reported in 36 (30 %) patients. Stomatitis (6 patients, 5 %), pneumonitis (5 patients, 4.2 %), pyrexia (4 patients, 3.3 %), aphthous stomatitis (3 patients, 2.5 %), and bronchitis (3 patients, 2.5 %) were the most common causes of dose adjustment or interruption. In the pediatric subpopulation, AEs requiring dose adjustment or interruption were reported in 27 (30.0 %) patients. The most commonly reported AEs in these patients were pneumonitis (5 patients; grade 3 in one patient), stomatitis (4 patients; grade 3 in two patients), pyrexia (4 patients; grade 3 in one patient), and bronchitis (3 patients; grade 3 in one patient). Other events were reported in ≤  2 patients.

Adverse events leading to hospitalization or prolonged hospitalization were reported in 29 (24.2 %) patients (Table 5). Of these, 23 were pediatric patients. Most commonly reported AEs were pyrexia (in four patients), bronchitis (in three patients), and gastroenteritis, viral gastroenteritis, pneumonia, sinusitis, diarrhea, stomatitis, hydrocephalus, status epilepticus, ovarian cyst (in two patients each).

Overall, SAEs were reported in 32 (26.7 %) patients. The most common SAEs were pyrexia (5 patients, 4.2 %) and bronchitis (3 patients, 2.5 %). Other SAEs reported (in >  1 %; two patients each) included diarrhea, gastroenteritis, viral gastroenteritis, hydrocephalus, ovarian cyst, pneumonia, pneumonitis, sinusitis, status epilepticus, stomatitis, urinary tract infection, and vomiting. Grade 3 SAEs were reported in 22 (18.3 %) patients and grade 4 in two (1.6 %) patients. Grade 4 SAEs were acute respiratory failure, aspiration pneumonia, and near drowning in one patient; gastroenteritis in the second patient.

Adverse events leading to study drug discontinuation were reported in eight (6.7 %) patients; four of them were pediatric patients. These AEs included increase in ƴ-GT (in two patients) and decreased white blood cell count, atrioventricular block, stomatitis, headache, hydrocephalus, somnolence, ovarian cyst, and respiratory tract inflammation (in one patient each).

Overall, in eight (6.7 %) patients, the hematology laboratory values worsened to CTC grade 3. These included low absolute neutrophils in six patients (4 patients in pediatric subpopulation), low hemoglobin in one patient (1 patient in pediatric subpopulation), and low white blood cells in one patient (none in pediatric subpopulation). None of the hematology values worsened to CTC grade 4. The biochemistry parameters most commonly reported with worsening to CTC grade 3 or 4 were low sodium in six patients (3 in pediatric subpopulation) and high ƴ-GT in five patients (2 in pediatric subpopulation). Two patients in the pediatric subpopulation had high potassium of grade 3.

No deaths occurred during the study or 28-day post treatment follow-up period and no significant changes were reported in vital signs from baseline to end of treatment.

### Efficacy

A partial response was reported in 81 (67.5 %) patients, a stable disease in 35 (29.2 %) patients, and a progressive disease in one (0.8 %) patient (Table 6). No patient had a complete response. The response was unknown in three (2.5 %) patients; in one patient, MRI was not required as per protocol because the patient completed the study before the first planned MRI assessment; in the second patient, there was a premature discontinuation for headache, hydrocephalus, and drowsiness; in the third patient MRI was not done, as the patient discontinued prematurely due to mother’s decision.

**Table 6:** Investigator assessed best overall response

Best response according to medical judgment	Everolimus (*N* = 120), *n* (%)
Complete response	0 (0.0)
Partial response	81 (67.5)
Stable disease	35 (29.2)
Progressive disease	1 (0.8)
Unknown	3 (2.5)

### Pharmacokinetics

Mean everolimus trough blood levels were below the target range of five to 15 ng/mL at week 2 (*n* = 109; 4.06 ng/mL; SD, 2.873) and at week 4 (*n* = 87; 4.89 ng/mL; SD, 2.937). The levels at week 6 (*n* = 57; 5.01 ng/mL; SD, 3.171) and at week 8 (*n* = 65; 5.27 ng/mL; SD, 3.011) were within the target range, although closer to the lower limit.

## Discussion

SEGAs have the potential to grow over time [6]. Active surveillance with neuroimaging is therefore recommended even for asymptomatic SEGAs. Surgical resection is the recommended treatment for acutely symptomatic SEGAs [17, 18]. For growing SEGAs that are asymptomatic, either surgery or mTOR inhibitors can be considered. Decision regarding the treatment is usually challenging and made in consultation with a multidisciplinary team that includes neurosurgeons, neurologists, neuroradiologists, and neurooncologists [17–19]. mTOR inhibitors have also been shown to be effective in the treatment of a number of other manifestations of TSC including renal angiomyolipoma, lymphangioleiomyomatosis, and epilepsy [20–27]. In patients with multisystem disease, treatment with mTOR inhibitors may therefore be preferred over surgery. The 2012 International TSC Consensus Conference guidelines for the surveillance and management of TSC recommend mTOR inhibitors for the patients with multisystem disease as well as for the patients with multiple or infiltrating tumors that are not amenable to surgical resection [18]. mTOR inhibitors may also be considered for shrinkage of a large tumor prior to surgical resection [28, 29].

EFFECTS sought to evaluate the safety and efficacy of everolimus for the treatment of patients with SEGA associated with TSC in a real-world setting. This expanded access program was initiated in 2011 to address an unmet need and make everolimus accessible to patients with SEGA associated with TSC, in countries where it was not yet commercially available. The inclusion criteria were specifically designed to enable maximum number of patients to participate and were therefore less restrictive than those for the open-label phase 1–2 study or the phase 3 EXIST-1 study. For example, diagnosis and follow-up of SEGA by either MRI or CT scan were allowed in this study.

The baseline characteristics of the disease observed in this study demonstrate the multisystem involvement in TSC. Majority of patients with SEGA also had other manifestations of TSC including other brain lesions (subependymal nodules and cortical tubers) and skin lesions (facial angiofibroma, forehead plaques, and hypomelanotic macules). In addition, renal angiomyolipomas were also seen in almost a half of the patients. It is important to note that modified Gomez criteria (1998) was used for the clinical diagnosis of TSC as the patient recruitment preceded the recommendations of the 2012 International TSC Consensus Conference.

The results of this expanded access program are comparable to the findings from previous studies of everolimus in SEGA associated with TSC. The AEs observed during this study were consistent with the known safety profile of everolimus in TSC [10, 11, 20, 30, 31]. Most AEs were of grade 1 or 2, and were manageable; no new safety concerns were identified. Aphthous stomatitis and infections continued to be the most common AEs as seen in the previous studies of everolimus treatment in TSC. In addition, the AEs observed in FAS and pediatric subpopulation appeared to be similar. Patients discontinuing due to drug-related AEs were a few. Interestingly, grade 3 or 4 AEs (23.3 %) appeared to be less frequent when compared to the phase 1–2 study where they were observed in 39 % of the patients [10]. In the EXIST-1 study, 33 % of the patients in the everolimus group vs 23 % in the placebo group experienced an AE of either grade 3 or 4 [11]. In the 2-year extension analysis, 51 % of the patients were reported to have grade 3 or 4 AEs [30]. The longer term data suggest that AEs seem to reduce over time and necessitates the need for an early detection and treatment of AEs [30, 31].

The effect of everolimus on growth and sexual maturation is not yet clear. In this study, of the 24 women with childbearing potential, three had amenorrhea and four reported irregular menstruation. While some data indicate that mTOR inhibition may partly be associated with the control of puberty onset [32], it has also been suggested that amenorrhea could be caused by TSC per se and may not be related to the treatment with mTOR inhibitors [33]. Following the report of amenorrhea in three of eight girls in EXIST-1, the protocol was amended to include long-term assessment of potential effects of everolimus on growth, development, and sexual maturation in the pediatric population [11]. In the 2-year extension data of EXIST-1 study, five of 28 women experienced amenorrhea; four of five cases resolved spontaneously [30]. In the EXIST-2 study, which evaluated the efficacy and safety of everolimus in the patients with angiomyolipoma associated with TSC or sporadic lymphangioleiomyomatosis, seven of 52 women treated with everolimus reported amenorrhea; four cases resolved; none of the patients discontinued treatment [20]. Long-term follow-up of these studies may give a clearer picture of the effect of everolimus on the reproductive system, if any.

Clinical benefit (partial response) was seen in majority (67.5 %) of the patients and a stable disease in about 30 % of the patients. Only one patient had disease progression. Moreover, these results were seen at a low normal range of everolimus blood levels. No conclusions could be drawn on pharmacokinetic parameters of everolimus as the blood samples collected in the EFFECTS study was aimed to maintain the mean everolimus trough blood levels within the desired range of 5–15 ng/mL. The efficacy of everolimus in TSC-associated SEGA has been established in previous studies. Efficacy was first demonstrated in the open-label, phase 1–2 study of 28 patients with serial growth of SEGA in which everolimus was associated with clinically relevant and statistically significant reduction in primary SEGA volume at 6 months relative to baseline (*P*  <  0.001) [10]. The long-term extension of this study demonstrated that everolimus was safe and effective for up to 3 years in these patients [31]. In the phase 3 EXIST-1 study (117 patients were randomized [2:1] to receive everolimus [*n*  =  78] or placebo [*n*  =  39]), 35 % of the patients in the everolimus group showed a minimum of 50 % reduction in the volume of SEGAs vs none in the placebo group (*P*  <  0.0001) [11]. The expanded access data from EFFECTS further support the efficacy and hence the use of everolimus in patients with SEGA associated with TSC.

## Conclusion

In summary, the results of this expanded access program confirm the acceptable safety profile of everolimus in patients <  18 years (pediatric subpopulation) and >  18 years of age, as shown by the low incidence of SAEs and grade 3 or 4 AEs. Reported events were consistent with the known safety profile of everolimus and manageable with the appropriate medical intervention. The results further support the efficacy of everolimus in the treatment of SEGA associated with TSC.

## Abbreviations

AEs, adverse events; CT, computed tomography; CTCAE, Common Terminology Criteria for Adverse Events; EFFECTS, Everolimus For Fast Expanded aCcess in TSC SEGA; EMA, European Medicines Agency; EXIST, EXamining everolimus In a Study of Tuberous sclerosis complex; FAS, full analysis set, MRI, magnetic resonance imaging; mTOR, mammalian target of rapamycin; SEGA, subependymal giant cell astrocytoma; TSC, tuberous sclerosis complex; USFDA, United States Food and Drug Administration
